# Effects of Intravenous Infusion of Iodine Contrast Media on the Tracheal Diameter and Lung Volume Measured with Deep Learning-Based Algorithm

**DOI:** 10.1007/s10278-024-01071-4

**Published:** 2024-03-06

**Authors:** Koichiro Yasaka, Hiroyuki Saigusa, Osamu Abe

**Affiliations:** Department of Radiology, Graduate School of Medicine, The University of Tokyo, 7-3-1 Hongo, Bunkyo-ku, Tokyo, 113-8655 Japan

**Keywords:** Contrast media, CT, Lung volume, Tracheal diameter, Deep learning

## Abstract

This study aimed to investigate the effects of intravenous injection of iodine contrast agent on the tracheal diameter and lung volume. In this retrospective study, a total of 221 patients (71.1 ± 12.4 years, 174 males) who underwent vascular dynamic CT examination including chest were included. Unenhanced, arterial phase, and delayed-phase images were scanned. The tracheal luminal diameters at the level of the thoracic inlet and both lung volumes were evaluated by a radiologist using a commercial software, which allows automatic airway and lung segmentation. The tracheal diameter and both lung volumes were compared between the unenhanced vs. arterial and delayed phase using a paired *t*-test. The Bonferroni correction was performed for multiple group comparisons. The tracheal diameter in the arterial phase (18.6 ± 2.4 mm) was statistically significantly smaller than those in the unenhanced CT (19.1 ± 2.5 mm) (*p* < 0.001). No statistically significant difference was found in the tracheal diameter between the delayed phase (19.0 ± 2.4 mm) and unenhanced CT (*p* = 0.077). Both lung volumes in the arterial phase were 4131 ± 1051 mL which was significantly smaller than those in the unenhanced CT (4332 ± 1076 mL) (*p* < 0.001). No statistically significant difference was found in both lung volumes between the delayed phase (4284 ± 1054 mL) and unenhanced CT (*p* = 0.068). In conclusion, intravenous infusion of iodine contrast agent transiently decreased the tracheal diameter and both lung volumes.

## Introduction

In the radiology department, contrast media is widely used. For CT and MRI examinations, iodine- and gadolinium-based contrast media, respectively, are used. However, the use of contrast media is associated with some adverse reactions. For both the iodine [1] and gadolinium-based contrast media [2], the most common major adverse reaction is hypersensitivity reaction. For patients with renal function impairment, radiologists need to exercise caution regarding contrast-induced nephropathy [3] and nephrogenic systemic fibrosis [4] from the iodine and gadolinium-based contrast media, respectively. For pregnant patients, gadolinium-based contrast media is associated with some adverse reactions including stillbirth or neonatal death [5]. Recently, two other new phenomena have been discovered: central nervous system cumulation of gadolinium for gadolinium-based contrast agent [6] and acute transient dyspnea after intravenous injection of gadoxetate disodium and gadobenate dimeglumine [7]. Additionally, we coincidentally found other new phenomenon associated with intravenous infusion of iodine contrast media, that is, a transient decrease in the tracheal diameter and lung volume in the arterial phase.

Airway diameter is associated with some diseases, especially asthma. In asthma, loss of homeostatic control of the airway smooth muscle causes hypercontractility with an increased risk for bronchospasm. A bronchodilator is used to manage this disease [8]. Additionally, a risk factor for adverse reactions in contrast agent use is poorly controlled bronchial asthma [9].

Owing to the deep-learning advancements in the field of radiology [10–12], an accurate evaluation of the lung volume became possible [13–15]. In the evaluation of several conditions, such as interstitial lung disease [16, 17], chronic obstructive pulmonary disease [18, 19], coronavirus pneumonia [20], size matching prior to lung transplantation [21], and detection of bronchiolitis obliterans in patients after lung transplantation [22], the utility of CT lung volumetry was reported in some studies.

Thus, fundamental data regarding the relationship between the infusion of iodine contrast agent vs. tracheal diameter and lung volume would be necessary. In the evaluation of this relationship, chest CT scans of both the unenhanced and contrast-enhanced CT performed within a single CT examination are ideal. In our hospital, vascular dynamic CT including chest, in which the unenhanced, arterial, and delayed phase are scanned in a single examination, satisfies this criterion. This study aims to investigate the effects of intravenous infusion of contrast material on the tracheal diameter and lung volume using chest dynamic CT images including unenhanced, arterial, and delayed phase performed within a single CT examination.

## Materials and Methods

Our Institutional Review Board approved this retrospective study, which waived the requirement for obtaining written informed consent from patients.

### Patients

**Fig. 1:**
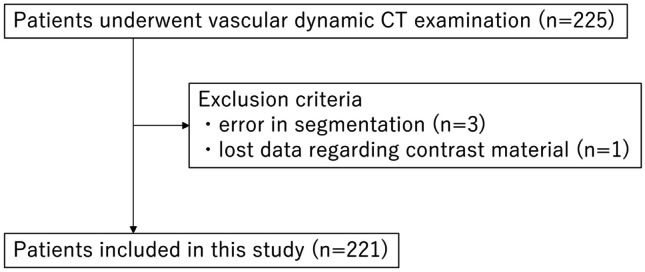
Flowchart for the patient inclusion and exclusion process

Patients who underwent vascular dynamic CT examination including the chest (unenhanced, arterial phase, and delayed phase) from January 2021 to February 2023 were included in this study. The following patients were excluded: (a) error in automatic segmentation of the lung or bronchus (*n* = 3) and (b) lost data regarding contrast material (*n* = 1). The patient inclusion and exclusion processes are illustrated in Fig. 1.

### CT Imaging

Patients underwent CT examination using CT scanners from two vendors (Canon Medical Systems [Tochigi, Japan] and GE Healthcare [WI, US]). Clinical indications for CT examination were the following: evaluations of abdominal aortic aneurysm (*n* = 109), peripheral arterial disease (*n* = 46), aneurysm of abdominal branches (*n* = 29), aortic dissection (*n* = 14), thoracic aortic aneurysm (*n* = 10), and others (*n* = 13). The CT scanning parameters were as follows: tube voltage at 120 kVp and tube current with automatic tube current modulation with standard deviation/noise index set at 13.0 for Canon-CT and 11.36 for GE-CT. The reconstruction parameters were the following: field of view which is adjusted to body size, slice thickness/interval of 1–1.25 mm/0.8 mm, and the kernel of FC04 for Canon-CT and STANDARD for GE-CT. Patients were instructed to hold deep-inspiratory level during the scan for all phases. These parameters were the same across unenhanced, arterial, and delayed phase.

The contrast agent was injected via the right or left antecubital vein within 30 s. The timing of the arterial phase imaging was determined through real-time monitoring of contrast enhancement by placing region of interest on the descending aorta at the level of the diaphragm. Scan was started when the CT attenuation of the aorta reached 250 Hounsfield unit. The delayed phase was scanned at 90 s after administration of the contrast agent injection.

### CT Image Evaluation

**Fig. 2:**
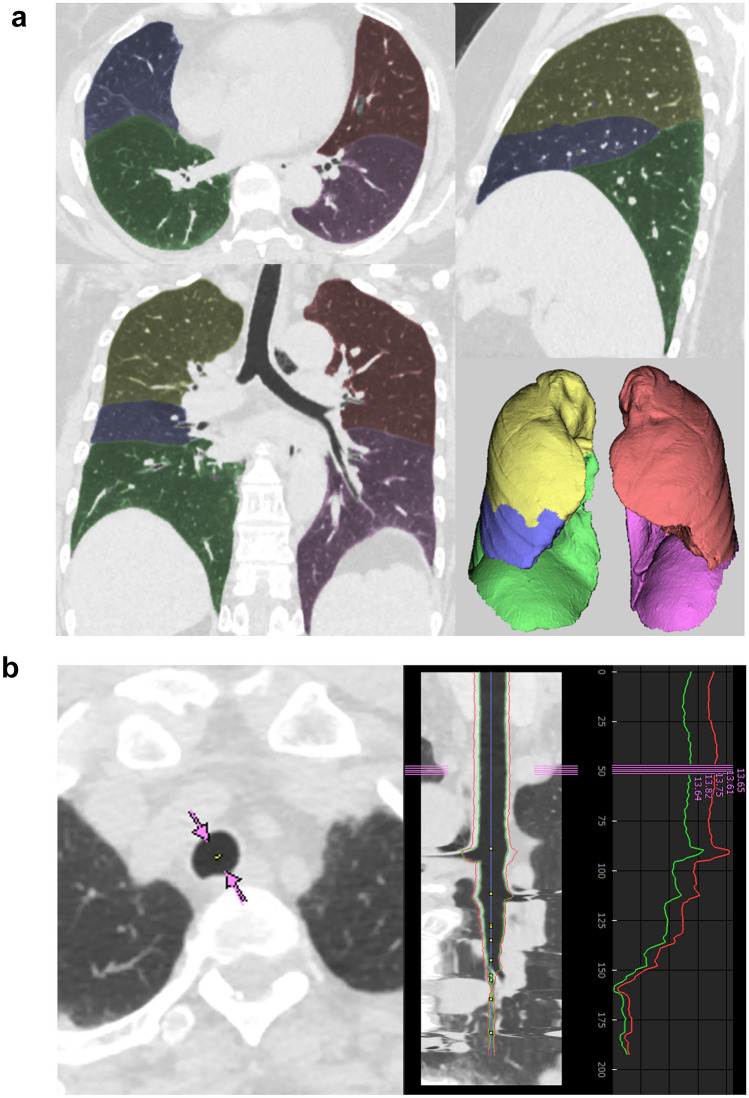
Evaluation of the **a** lung volume and **b** tracheal diameter using a software. **a** The right upper lobe, right middle lobe, right lower lobe, left upper lobe, and left lower lobe were segmented and highlighted with yellow, blue, green, red, and pick, respectively. **b** Trachea at the thoracic inlet is annotated with pink arrows in the left CT image. Inner lumen and outer boundary are shown with green and red lines, respectively. Inner lumen diameter is also displayed as pink numerals

A radiologist with a 13-year diagnostic imaging experience performed the CT image evaluation using a commercial software (Synapse Vincent, Fujifilm [Tokyo, Japan]). This software, which was developed based on the deep-learning algorithm, allowed segmentation of each lobe of the lung as well as the entire lung fully automatically (Fig. 2a). The radiologist recorded the CT lung volume for each lobe including both lung volumes. Then, automatic segmentation of the airway was also performed. With this software, the inner lumen diameter of the trachea, which is averaged for the long axis and short axis, is displayed (Fig. 2b). The averaged tracheal diameter at the thoracic inlet was recorded.

The arterial phase to the unenhanced and delayed phase to the unenhanced ratio for the tracheal diameter (TD_AU_ and TD_DU_, respectively), both lung volumes (BLV_AU_ and BLV_DU_, respectively), and each lobe volume were calculated.

### Statistical Analysis

Statistical analysis was performed using R version 4.1.2 (https://www.r-project.org/). The tracheal diameter and both lung volumes were compared between the unenhanced CT vs. the arterial phase and unenhanced vs. delayed phase using the paired *t*-test. The Bonferroni correction was performed for these analyses because multiple groups were compared. Correlations between the patient’s age vs. the tracheal diameter and both lung volumes were assessed using the Pearson’s correlation coefficient. Associations between background factors vs. the tracheal diameter and both lung volumes were assessed using a Student’s *t*-test or analysis of variance. Statistical significance was set at a *p* value < 0.05.

## Results

### Patients

**Fig. 3:**
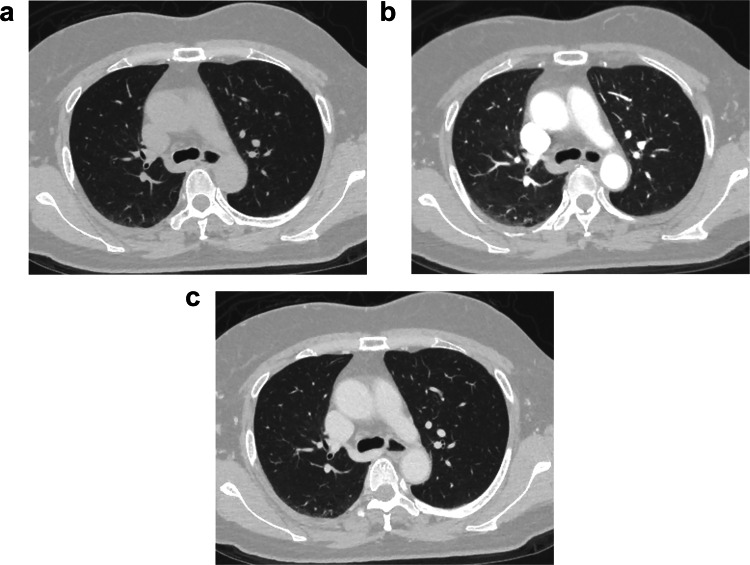
Representative CT image of a 61-year-old female patient who was administered with an iodine contrast agent (iopamidol, 370 mgI/mL) from the right antecubital vein. Both lung volumes in the **a** unenhanced, **b** arterial phase, and **c** delayed phase images were 2144, 1739, and 2434 mL, respectively. The tracheal diameters at the thoracic inlet in the unenhanced, arterial phase, and delayed phase were 13.6, 12.6, and 14.3 mm, respectively

Patient background information is described in Table 1. A total of 221 patients (71.1 ± 12.4 years, 174 males) were included. Representative vascular dynamic CT images are shown in Fig. 3.

**Table 1:** Patient background information

	Value
Number of patients	221
Age (years)	71.1 ± 12.4
Sex	
Male	174
Female	47
Generic name of contrast material	
Iomeprol	21
Iopamidol	129
Iohexol	57
Ioversol	14
Iodine density of contrast material	
≤ 350 mgI/mL	101
> 350 mgI/mL	120
The side of injection	
Left	36
Right	185
CT scanner	
Canon-CT	217
GE-CT	4

### Tracheal Diameter and Lung Volume Comparisons Between Each Phase

**Fig. 4:**
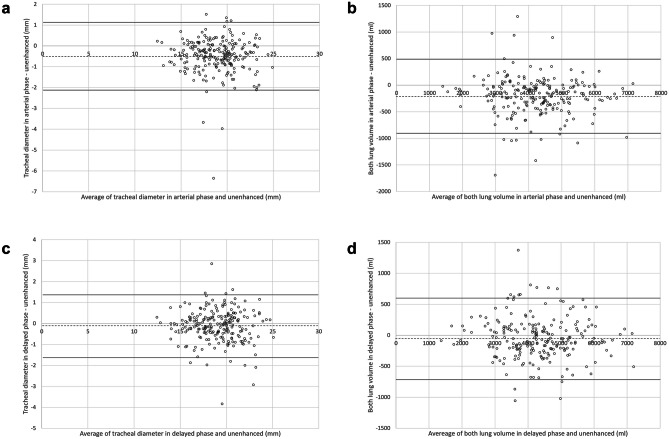
Bland–Altman plots for the **a** tracheal diameter between the arterial and unenhanced, **b** both lung volumes between the arterial and unenhanced, **c** tracheal diameter between the delayed phase and unenhanced, and **d** both lung volumes between the delayed phase and unenhanced. Dashed and solid lines indicate mean of difference and limits of agreement, respectively

**Fig. 5:**
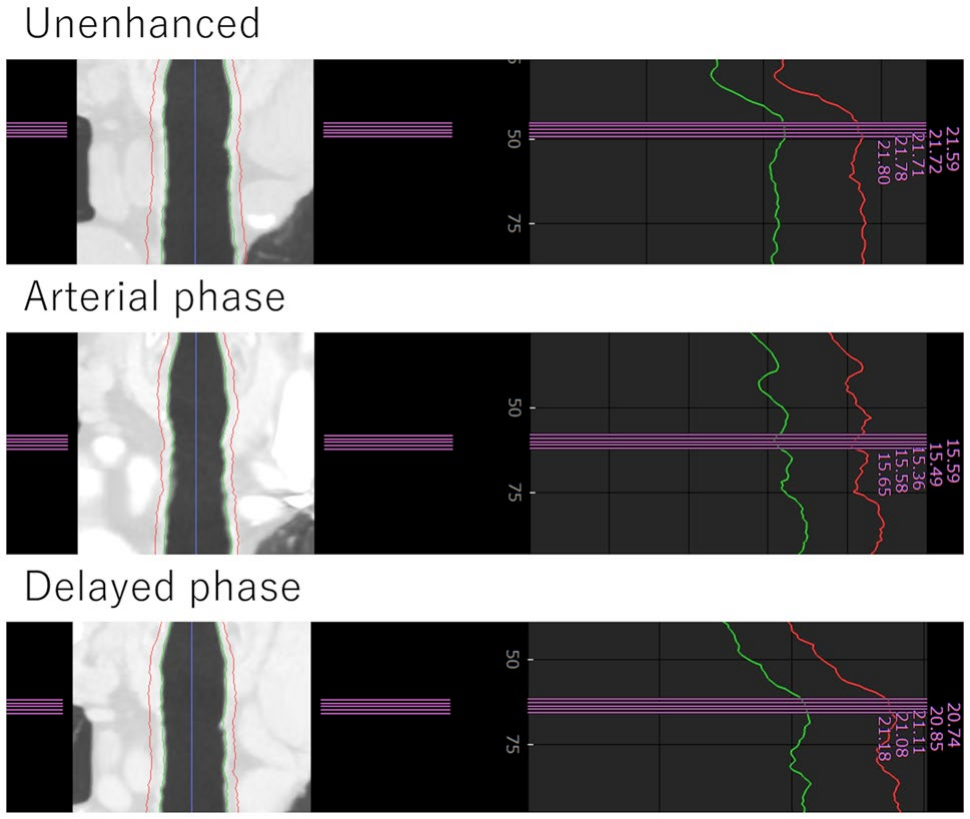
Representative CT images (left) and tracheal diameter data images (right) of an 85-year-old male patient in whom the difference of the tracheal diameter between unenhanced (21.7 mm) and arterial phase (15.4 mm) was the largest. There was no considerable misregistration for the trachea

The detailed results of the tracheal diameter and lung volume are provided in Table 2. Bland–Altman plots are shown in Fig. 4. The tracheal diameter in the arterial phase was 18.6 ± 2.4 mm which was significantly smaller than those in the unenhanced CT (19.1 ± 2.5 mm) (*p* < 0.001). Even when the outliers with a difference > 3 mm as seen in Fig. 4a were excluded, the average tracheal diameters in unenhanced (19.1 mm) and arterial phase (18.6 mm) have not changed, and there was still a statistically significant difference between them (*p* < 0.001). There was no considerable misregistration by the software for these patients (Fig. 5). No statistically significant difference was found in the tracheal diameter between the delayed phase (19.0 ± 2.4 mm) and unenhanced CT (*p* = 0.077).

**Table 2:** Tracheal diameter and both lung volumes in each phase

	Unenhanced	Arterial phase	Delayed phase
Tracheal diameter (mm)	19.1 ± 2.5	18.6 ± 2.4	19.0 ± 2.4
Lung volume (mL)			
Both lungs	4332 ± 1076	4131 ± 1051	4284 ± 1054
Right lung	2383 ± 578	2282 ± 565	2360 ± 569
Right upper lobe	966 ± 256	935 ± 249	958 ± 251
Right middle lobe	431 ± 142	425 ± 143	433 ± 141
Right lower lobe	987 ± 349	922 ± 334	969 ± 341
Left lung	1949 ± 533	1849 ± 520	1924 ± 522
Left upper lobe	1147 ± 286	1107 ± 285	1138 ± 282
Left lower lobe	802 ± 314	742 ± 299	786 ± 306

Mean ± standard deviation is provided

Both lung volumes in the arterial phase were 4131 ± 1051 mL which was significantly smaller than those in the unenhanced CT (4332 ± 1076 mL) (*p* < 0.001). Conversely, no statistically significant difference was found in the volume of both lungs between the delayed phase (4284 ± 1054 mL) and unenhanced CT (*p* = 0.068).

Data regarding the TD_AU_, TD_DU_, BLV_AU_, and BLV_DU_ values are described in Table 3. The tracheal diameter and both lung volumes in the arterial phase were decreased by 2.5% and 4.4%, respectively, compared with the unenhanced CT. In the arterial phase, a more than 10% decrease in the tracheal diameter was observed for 1.8% of patients. The lung volume was decreased in more than 10% in 18.1% of patients.

**Table 3:** Percentage of patients based on the arterial phase to unenhanced ratio and delayed phase to unenhanced ratio for the tracheal diameter and both lung volumes

	TD_AU_	TD_DU_	BLV_AU_	BLV_DU_
Average	0.975 ± 0.042	0.995 ± 0.040	0.956 ± 0.091	0.993 ± 0.085
< 1.00	74.2% (164/221)	54.3% (120/221)	77.4% (171/221)	57.0% (126/221)
< 0.90	1.8% (4/221)	1.8% (4/221)	18.1% (40/221)	11.3% (25/221)
< 0.80	0.5% (1/221)	0.0% (0/221)	3.6% (8/221)	0.9% (2/221)
> 1.00	25.8% (57/221)	45.7% (101/221)	22.6% (50/221)	43.0% (95/221)
> 1.10	0.0% (0/221)	0.5% (1/221)	2.7% (6/221)	9.5% (21/221)

Mean ± standard deviation is provided for the average

*BLV*_*AU*_ arterial to unenhanced ratio for both lung volumes, *BLV*_*DU*_ delayed to enhanced ratio for both lung volumes, *TD*_*AU*_ arterial to unenhanced ratio for the tracheal diameter, *TD*_*DU*_ delayed to unenhanced ratio for the tracheal diameter

Significant positive correlations exist between TD_AU_ vs. BLV_AU_ (*r* = 0.533 [95% confidence interval, 0.432–0.622], *p* < 0.001) and TD_DU_ vs. BLV_DU_ (*r* = 0.464 [95% confidence interval, 0.353–0.561], *p* < 0.001).

### Arterial to Unenhanced and Delayed to Unenhanced Ratios of Each Lobe Volume

The arterial phase to unenhanced ratio for the right upper lobe, right middle lobe, right lower lobe, left upper lobe, and left lower lobe was 0.971 ± 0.075, 0.985 ± 0.076, 0.941 ± 0.135, 0.966 ± 0.076, and 0.931 ± 0.137, respectively. The volume of the right lower lobe and left lower lobe, which is located near the diaphragm, was more largely affected by the intravenous infusion of contrast material than the upper and middle lobes.

The delayed phase to unenhanced ratio for the right upper lobe, right middle lobe, right lower lobe, left upper lobe, and left lower lobe was 0.995 ± 0.063, 1.010 ± 0.053, 0.992 ± 0.134, 0.994 ± 0.065, and 0.991 ± 0.135, respectively.

### Factors Affecting the Arterial to Unenhanced Ratio for the Tracheal Diameter and Lung Volume

The detailed results for the association between each background factor vs. TD_AU_ and BLV_AU_ are described in Table 4. The right-side injection was found to be significantly associated with a lower BLV_AU_ value (0.950 ± 0.091) compared with the left-side injection (0.988 ± 0.086) (*p* = 0.022). No significant factor was found to affect the TD_AU_. The patients’ age, sex, and type or iodine concentration of the contrast material did not have a significant impact on the TD_AU_ or BLV_AU_ values (*p* > 0.300).

**Table 4:** Association between factors and arterial phase to unenhanced ratio for the lung volume and tracheal diameter

	TD_AU_		BLV_AU_	
	Value	*p* value	Value	*p* value
Age	− 0.064 (− 0.195–0.0683)	0.342	− 0.034 (− 0.165–0.0987)	0.618
Sex		0.472		0.300
Male	0.976 ± 0.044		0.960 ± 0.086	
Female	0.971 ± 0.033		0.944 ± 0.107	
Contrast material		0.529		0.739
Iomeprol	0.982 ± 0.033		0.960 ± 0.063	
Iopamidol	0.973 ± 0.048		0.951 ± 0.086	
Iohexol	0.975 ± 0.029		0.966 ± 0.112	
Ioversol	0.987 ± 0.037		0.960 ± 0.078	
Iodine concentration		0.673		0.471
≤ 350 mgI/mL	0.974 ± 0.045		0.961 ± 0.108	
370 mgI/mL	0.976 ± 0.038		0.952 ± 0.074	
The side of injection		0.396		0.022*
Left	0.980 ± 0.037		0.988 ± 0.086	
Right	0.974 ± 0.043		0.950 ± 0.091	

For age, correlation coefficients (95% confidence interval) are provided

Comparisons were performed using Student’s *t*-test or analysis of variance

*BLV*_*AU*_ arterial phase to unenhanced ratio for both lung volumes, *TD*_*AU*_ arterial phase to unenhanced ratio for the tracheal diameter

*Statistically significant

## Discussion

Allergic reaction and contrast-induced nephropathy are well-known adverse reactions associated with iodine contrast material. Additionally, we found and confirmed a new phenomenon: a transient decrease in the tracheal diameter and lung volume. The tracheal diameter and both lung volumes were decreased by 2.5% and 4.4%, respectively, in the arterial phase, and a more than 10%/20% decrease of tracheal diameter and both lung volumes was observed in 1.8%/0.5% and 18.1%/3.6% of patients, respectively. These phenomena were transient, and no significant effect was observed in the delayed phase.

There have been studies which reported factors affecting the diameter or airway volume. Yamada et al. reported that the ratio of inspiratory to expiratory airway volume in supine position is larger than the standing position in patients with chronic obstructive pulmonary disease (*p* < 0.001) [19]. In asthmatics, for whom the bronchoalveolar lavage fluid count tend to be high, the eosinophil count in the bronchoalveolar lavage fluid was negatively associated with the airway diameter (*r* =  − 0.7, *p* < 0.05) [23]. Additionally, while obesity is known to be associated with a higher incidence and prevalence of asthma [24], abdominal visceral fat area was negatively associated with airway diameter in asthmatics (*r* =  − 0.35, *p* = 0.01) [25]. Furthermore, the barometric pressure has an impact on the increased risk for hospital visit for asthma [26]. Our study is unique in that the association between contrast agent injection, which is known to be a risk factor for patients with poorly controlled asthma [9], and decrease in tracheal diameter was demonstrated. Whether this phenomenon can be a trigger for hypersensitivity reaction in patients with or without asthma requires further investigation.

Chest CT examination is sometimes performed at levels other than end-inspiratory level. One representative case is CT pulmonary angiography, which is sometimes scanned at mid-inspiratory level to reduce the risk of transient interruption of contrast [27]. However, chest CT image scanned at mid-inspiratory level is known to increase a risk of nondiagnostic lung images [27]. From our study, it was found that more than 10% or 20% decrease of both lung volumes was observed in 18.1% and 3.6% of patients, respectively. In daily clinical practice, it would be better to avoid scanning chest at the arterial phase when aimed to evaluate the lung at the end-inspiratory level.

In interstitial lung diseases, it has been reported that both lung volumes were 3345 mL, and the relative annual bilateral lung volume loss was reportedly 2.07% and 17.44% in patients without and with major adverse event at a 3-year follow-up, respectively [17]. In our study, it was found that both lung volumes decreased by an average of 4.4% for the arterial phase compared with the unenhanced CT, and more than 10% and 20% decrease was observed in 18.1% and 3.6% of patients, respectively. The effect of a transient decrease in both lung volumes in the arterial phase would not be negligible in the lung volume assessment of interstitial lung disease.

The ratio of the expiratory volume to inspiratory volume in the lower lobes has been reportedly smaller than those in the upper and middle lobes (41.1–41.7% vs. 51.9–65.4% [28] or 57.4–57.8% vs. 67.5–74.1% [19]). This would be attributed to the fact that the lower lobes are located near the diaphragm which play an important role in respiration. In the current study, the volume of the lower lobes was more largely decreased (93.1–94.1%) in the arterial phase compared with the upper or middle lobes (96.6–98.5%). This fact indicates that the lobe volume decrease in the arterial phase was caused by the movement of the diaphragm. In addition, not only the lung volume but also the tracheal diameter was decreased in the arterial phase, and significant correlation between TD_AU_ and BLV_AU_ was observed. Furthermore, we excluded patients in whom errors in segmentation of the lung or bronchus were seen. These facts suggest that the change in the arterial phase was caused by the respiration not merely by the segmentation performance variation caused by the CT attenuation of the pulmonary vessels.

The right-side injection was significantly associated with a lower BLV_AU_ value (0.950) compared with the left-side injection (0.988) (*p* = 0.022). This may be associated with the fact that the length between the antecubital vein and the right side of the heart is shorter for the right antecubital vein. A transient dilatation of right side of the heart or transient chemical reaction caused by the contrast agent molecule may be possible reasons for this phenomenon; however, a detailed mechanism remains unknown.

This study has some limitations. First, the relationship between the hypersensitivity reaction secondary to the contrast agent and the tracheal diameter was not assessed in this study. Future study regarding this topic is warranted. Second, because a CT scan was performed during end-inspiration level, whether change could be observed even in resting breathing state or patients just experienced difficulty in deep inspiration remains unclear. Third, because patients included in this study did not necessarily have lung diseases, depiction of diseases was not assessed. Finally, all patients included in this study underwent vascular dynamic CT examinations. Future studies including patients with other conditions are warranted.

In conclusion, the tracheal diameter and both lung volumes were transiently decreased in the arterial phase of vascular dynamic CT examination compared to unenhanced CT. To reveal the onset and duration of this phenomenon, future studies including CT examinations other than vascular dynamic CT is needed. In addition, the mechanism of this phenomenon is needed to be investigated in future studies.

## Abbreviations

BLV_AU_: Arterial phase to unenhanced ratio for both lung volumes
BLV_DU_: Delayed phase to unenhanced ratio for both lung volumes
TD_AU_: Arterial phase to unenhanced ratio for the tracheal diameter
TD_DU_: Delayed phase to unenhanced ratio for the tracheal diameter
